# Multidisciplinary Simulation Improves Resident Confidence for Pregnant Patients Requiring Surgical Intervention

**DOI:** 10.7759/cureus.23454

**Published:** 2022-03-24

**Authors:** James Harrington, Gary Duncan, Karen DAngelo, Brad D Gable

**Affiliations:** 1 General Surgery, Cleveland Clinic, Cleveland, USA; 2 Medical Education and Simulation, OhioHealth, Columbus, USA; 3 Graduate Medical Education, OhioHealth, Columbus, USA; 4 Simulation, Riverside Methodist Hospital/OhioHealth, Columbus, USA

**Keywords:** multidisciplinary team, emergent general surgery, emergency obstetrics, hepatocellular adenoma, 3d printing, simulation in medical education

## Abstract

Introduction: Hepatocellular adenomas are a rare but serious cause of bleeding, which is further complicated by pregnancy. Interprofessional cooperation is a key component of residency education, thus simulations designed to integrate multiple programs are mutually beneficial. This simulation details surgical and obstetric management of a pregnant patient in hemorrhagic shock from a bleeding hepatocellular adenoma. Objectives for the study were to evaluate learners’ confidence to 1) prioritize the care of a pregnant patient with hemoperitoneum and hemorrhagic shock, 2) demonstrate interdisciplinary collaboration with other specialties, 3) apply massive transfusion protocol (MTP) in the appropriate clinical setting, and 4) analyze critical decisions for evaluating pregnant females with severe abdominal pain.

Methods: Obstetric, general surgery, and anesthesia residents, along with labor and delivery nurses participated in a simulated clinical scenario that focused on the management of a pregnant patient in hemorrhagic shock. The learners evaluated the educational session using a standard Return on Investment in Learning survey immediately following the session.

Results: A total of 23 residents and medical students gave feedback on the experience. The main learning objectives were met with increased confidence in the four learning objectives by 77.3-95.4% of responders. Overall, greater than 90% of participants felt the simulation was relevant to their training and realistic, with 100% responding that the course provided new, or clarified existing information for them.

Conclusion: A multidisciplinary simulation-based educational intervention was successful in improving learner confidence in managing a complicated surgical emergency in a pregnant patient with inter-residency cooperation.

## Introduction

Hepatocellular adenomas (HCA) are rare, benign lesions of the liver that are associated with estrogen use. Although their association with oral contraceptive (OC) use is established, it is uncommon, being seen in approximately 30 cases per one million OC users [1,2]. Due to the potential growth with increased estrogen exposure and risk of rupture, cessation of OCs is often the first-line treatment for HCA [3]. With the cessation of exogenous estrogen, HCA resolution can be seen. However, HCAs can recur with resumption of OCs or pregnancy [4]. Due to the elevated risks of rupture in pregnancy, some have suggested management of adenomas >5 cm in size prior to pregnancy [5,6].

Due to the rarity of HCA, the incidence of intraoperative hemorrhage secondary to ruptured HCA in pregnancy is exceedingly rare. However, it has been documented. In a recent case report, a 30-year-old primigravid was managed with temporary packing and definitive control of hemorrhage via angioembolization of a bleeding HCA [7]. This case report highlighted the importance of a multidisciplinary approach in managing this rare, but life-threatening condition. We have previously described multidisciplinary education involving severely injured pregnant trauma patients [8]. We sought to evaluate a different low-frequency high-risk situation with this scenario. Content experts from obstetrical-gynecology (OB/Gyn) and general surgery (GS) determined that at our institution there are several cases per year where a pregnant patient is taken urgently/emergently to the labor and delivery operating room for operative delivery and is then found to have pathology such as hemoperitoneum. While there are numerous causes of hemoperitoneum and shock in pregnant females, we felt HCA provided new opportunities for our learners.

The use of simulation to improve communication, teamwork, and patient care has been well documented [9-11]. In this simulation-based education, we sought to establish a multidisciplinary scenario between anesthesiology, OB/Gyn, and GS residencies at a single institution where teams encountered massive hemorrhage secondary to a ruptured HCA at the time of cesarean section. Our goal was to create a realistic simulation that allowed for evaluation of effective communication, intraoperative mechanical control of hemorrhage, appropriate intraoperative resuscitation, and definitive control of hemorrhage of this rare but life-threatening condition.

## Materials and methods

Learner population

Ob/Gyn, GS, anesthesia residents, and labor and delivery nurses all participated in the simulation education. A convenience sample was taken of those residents and nurses that were able to participate during the simulation time.

Educational session description

A simulation case was developed where OB/Gyn residents were asked to evaluate a hemodynamically unstable pregnant patient in the emergency department. The assessment included evaluating a gravid standardized patient who was representing a primigravida patient at 34 weeks and 0 days gestation that had sudden onset of severe abdominal pain with rebound and guarding. The case started in our simulated emergency room as this would be where this patient would be triaged based on institutional protocols. Since the focus of this case was on intraoperative decisions and communication, we did not involve learners from the emergency department. Assessment of the fetus in the emergency department showed a heart rate of 130 beats per minute with moderate variability and an increasing number of late decelerations. After assessing the patient, the decision was made to take the patient emergently to the simulated labor and delivery operating room to perform cesarean delivery of the fetus.

A Noelle ®(Gaumard Scientific Company, Inc., , Miami, FL, USA) mannequin that was no longer in use was utilized for the cesarean delivery portion of the case. The abdominal cavity was emptied of all its mechanical and electrical hardware and lined with waterproof plastic bags. The gravid uterus was recreated using a balloon that contained the Noelle ® fetus and water, and this was contained within foam and pantyhose.

The liver model was created using silicone foam with silicone skinning techniques. A liver with the ability to exsanguinate was required for the case and our simulation team was unable to identify a commercially available product. Therefore, the OhioHealth Simulation Team developed a novel model. Through the NIH 3D Print Exchange, a 3D scan of a liver was acquired. That file was placed in Blender (3D modeling software) and 3D printed using a Prusa MK3 brand printer and polylactic acid (PLA) filament with Soma Foama and silicone. This model was then converted to a negative mold design to build the liver. Within the mold, Smooth-On Soma Foama silicone foam was poured. The mold was then skinned with Smooth-On Dragon Skin and the liver was recast to coat with a durable layer of silicone. When the liver was complete, a needle driver was used to bore a hole and insert IV tubing to carry the “blood”. Additionally, a remote-controlled pump was manufactured that delivered blood from an IV bag to the hole in the liver. This allowed controlled blood flow from the simulation control room with no interruption to the learners. Total cost of materials to generate this model was $80. Total time to 3D print the liver models was 20 hours, and an additional 6 hours of 3D printing for the pump housing. Total time for assembly of the model was 17 hours.

Upon entering the abdomen of the simulator, the learners encountered a large amount of blood. The patient became hypotensive and tachycardic. As the OB/Gyn residents delivered the fetus, the anesthesia residents worked to resuscitate the mother. This included initiating the massive transfusion protocol (MTP). In addition, GS residents were contacted and came emergently to the operating room. The GS residents performed a damage control laparotomy and identified the source of hemorrhage as an HCA and mitigated the bleeding accordingly.

The education was planned on a day when OB/Gyn and GS residents share didactic time. The simulation case was run two different times, and each case included three OB/Gyn, three GS, and two anesthesia residents. Some GS and anesthesia residents participated in both simulation cases (each resident only completed 1 post-simulation survey). All other learners were able to watch the simulation and participated in the debrief that followed each of the simulation sessions. Each simulation case took approximately 15 minutes, and each debrief took approximately 55 minutes. The debriefing was facilitated by simulation/debriefing experts with contributions from OB/Gyn, GS, and anesthesia content experts. The debriefing followed the 4E format (Emotions, Events, Empathy, Explanations). Learning objectives for the session were: by the end of this session, learners will be able to (1) prioritize the care of a pregnant patient with hemorrhagic shock from hemoperitoneum, (2) demonstrate interdisciplinary collaboration with other specialties, (3) apply MTP to the appropriate clinical settings, and (4) analyze critical decisions made for abdominal pain in pregnant female patients.

Educational evaluation

A standardized Return on Investment in Learning (ROL) assessment tool, based on John Phillips’ Return on Investment, was used to determine participants’ reactions to and application of the educational intervention [12]. These evaluations are part of our standard ROL assessment developed by OhioHealth Learning that is conducted for all simulation activities. These ROL levels are Level 0-Inputs (Number of learners); Level 1-Reaction (Preference Needs); Level 2-Learning (Learning Needs); Level 3-Application (Performance Needs); Level 4-Impact (Business Needs); Level 5-ROI (Payoff Needs).

The ROL framework is used as a feedback mechanism for educators to determine which areas of education are the most beneficial, and where educators might be able to improve the education in the future. The evaluation measures for this event meet levels 0-2 based on the assessment below. The survey, provided at the conclusion of the debrief of each simulation, consisted of 20 questions detailing demographics, learning objective effectiveness by 5-point Likert scale, and open-ended questions to capture additional feedback. Objectives were also rated on perceived difficulty. This project was reviewed by the OhioHealth Institutional Review Board and did not meet the criteria for human subjects research, but was qualified as a quality improvement project.

Statistical analyses

The statistical summary for this project involves descriptive statistics of the simulation survey. Statistical data was analyzed using standard deviation and percentage-based calculations by the research team through Social Science Statistics T and P-value calculator [13].

## Results

For this education, 23 participants responded to the post-simulation questionnaire, representing our ROL Level 0 data. Completed participant questionnaires were obtained from three GS residents (one GS resident started the questionnaire but did not complete any responses due to clinical obligations), 12 OB/Gyn residents, three anesthesia residents, three medical students completing their core rotations, and one obstetrical surgical technician who assisted with the procedure. Additional demographic information relating to sex, gender, age, or other information was not obtained for this study.

Objectives measured for this simulation included self-reported improvement in participant confidence. The first objective entailed improved confidence in “my ability to prioritize the care of a pregnant patient with hemorrhagic shock from hemoperitoneum.” Twenty-one of 22 respondents either agreed or strongly agreed with this statement (95.4%). Twenty-one of 22 (95.4%) participants agreed or strongly agreed with the statement, “I feel more confident in my ability to demonstrate interdisciplinary collaboration with other specialties.” Seventeen participants (77.3%) agree/strongly agree that they felt more confident in their ability to apply MTP to the appropriate clinical setting. In addition, participants felt more confident in their ability to analyze critical decisions made for abdominal pain in pregnant female patients (90.9%) (Table 1).

**Table 1:** Post-simulation learner self-reported confidence of the learning objectives

Objectives: “I felt more confident in my ability to …”	Agree/Strongly Agree (%)
1) Prioritize the care of a pregnant patient with hemorrhagic shock from hemoperitoneum	95.4
2) Demonstrate interdisciplinary collaboration with other specialties	95.4
3) Apply massive transfusion protocol (MTP) to the appropriate clinical setting	77.3
4) Analyze critical decisions made for abdominal pain in pregnant female patients	90.9

**Figure 1:**
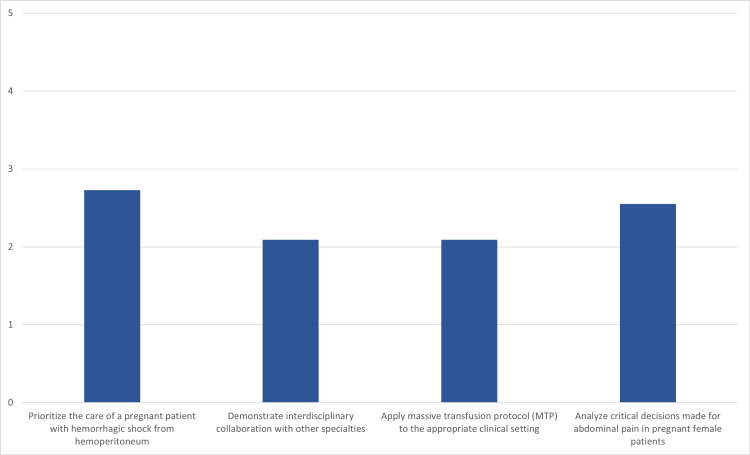
Post-simulation learner self-reported perceived difficulty with performing the objectives Scale: 1—not difficult; 2—somewhat difficult; 3—difficult; 4—very difficult; 5—extremely difficult

On a five-point Likert scale from not difficult to extremely difficult, participants rated key activities covered in the simulation. Weighing the scales from one (not difficult) to five (extremely difficult), the participants rated the prompts in the following order from least to most difficult: apply MTP to appropriate clinical settings (2.09), demonstrate interdisciplinary collaboration with other specialties (2.09), analyze critical decisions made for abdominal pain in pregnant female patients (2.55), and prioritize the care of a pregnant patient with hemorrhagic shock from hemoperitoneum (2.73). This characterization of data showed the greatest clarity in perceived difficulty in the simplest manner, rather than highlighting agree/strongly agree as in Table 1. Because the objectives were rated on a sliding scale of difficulty instead of agreement, an overall average provided the clearest picture of the participants’ responses (Figure 1).

Participants were asked about the relevance of the simulation to their work and 20/22 strongly agreed and 2/22 agreed, giving 100% positive feedback for course relevance. All 22 participants (100%) also agreed/strongly agreed that the simulation provided new, or clarified existing, information, that they intended to use what they learned from the simulation, that the facilitators were knowledgeable about the subject, and that the environment was conducive to learning. Nearly all learners, 95.4% (21/22) agreed/strongly agreed that the simulation was realistic and that the instructors were responsive to participants’ needs and questions.

## Discussion

Interdisciplinary simulations have proven to be beneficial for resident learning and resident attitudes toward other specialties with whom they work [9,14,15]. This simulation allowed multidisciplinary providers to engage in collaborative learning while highlighting a low-frequency high-risk situation. These scenarios are ideal for cooperative simulation-based learning [16]. This simulation-based education provided a unique opportunity to utilize decision-making not only for the mother but also for the fetus and doing so in a multidisciplinary manner. The stated goals of the simulation included inter-specialty collaboration as well as specific medical competencies that are crucial to anesthesiology, surgical, and OB/Gyn residents. Accreditation Council for Graduate Medical Education (ACGME) common program requirements include “working in interprofessional teams to enhance patient safety and improve patient care quality [17].”

Bloom’s Taxonomy revised action verbs were used in the objectives to ensure actionable and measurable outcomes for the simulation [18]. Due to the infrequent nature of the specific case portrayed, patient-centered outcomes related to the education were not able to be captured. ROL was used to evaluate the participants’ response to the curriculum and self-reported efficacy of the training.

Overwhelmingly, participants felt improved confidence in all aspects of the objectives laid out for the education. Multiple participants specifically commented on the benefit of having the debrief session be multidisciplinary because it allowed participants to understand different perspectives and priorities during the case. This was highlighted by one OB/Gyn resident stating that the most helpful portion of the course was, “Hearing general surgery’s recommendations for what is helpful when they aren’t immediately available… packing all four quadrants of the abdomen.”

The ability to prioritize care of a pregnant patient with hemorrhagic shock from hemoperitoneum was rated by participants as the most difficult objective (2.73/5) of the case using the post-simulation Likert scale. It also had the highest agreement in improving the participants’ confidence (21/22) after completing the education. This is encouraging as the residents found this to be a difficult concept but felt much better about the subject at the completion of the education. The objective with the lowest percentage of increased confidence after the course was the ability to apply MTP (17/22). This was also rated as the easiest concept on the difficulty scale (2.09/5). This inverse relationship compared to the previous example may indicate a higher level of initial confidence in executing this protocol, so the simulation did not further enhance this knowledge for some participants. Overall, however, each of the objectives had a significant majority of participants report increased confidence in the objectives being measured.

Over 95% of participants rated the simulation as realistic and included a comment stating, “Amazing sim to be able to do the operative case in real time!” There were two comments about the improvement of the model for increased realism for future simulations. One participant reported a desire for the patient to not be fully draped at the beginning of the surgical case, which decreased realism of the scenario. Another commented that the amount of hemorrhage was difficult to estimate based on the bleeding from the 3D printed model. The comment stated, “Sim model was great-just need some visual feedback on the amount of hemorrhage.” An additional suggestion for improvement of the course was provided by a medical student who requested a printed handout of the objectives and takeaways for additional study at the conclusion of the course.

The technical aspects of utilizing 3D printing to create a realistic liver model with mechanical blood flow were essential in achieving the realism needed for this course. 3D printing allows for low-cost, highly realistic models to be used in effective medical simulation to enhance medical education [19,20].

Limitations of the study include lack of follow-up data to establish long-term retention of knowledge gained, and the ability of learners to apply this knowledge. As mentioned above, being a rare patient encounter, patient-specific data was unable to be obtained. There was a relative imbalance of OB residents to surgical and anesthesiology residents who responded to the survey, which could have influenced the data toward learning outcomes that affected OB/Gyn more specifically. As an independent academic medical center, multiple surgical specialty residency programs were available to participate thanks to a shared didactic time which may be difficult to replicate in other settings.

## Conclusions

A multidisciplinary simulation-based education successfully improves participant confidence in managing a pregnant patient in hemorrhagic shock requiring additional resuscitation and surgical intervention. A novel 3D printed model of a liver HCA was realistic and well-liked by learners. Multidisciplinary simulations enhanced with 3D printing technologies increase learners’ confidence and experience of low-frequency high-risk scenarios.

## References

[1] Rooks JB, Ory HW, Ishak KG, Strauss LT, Greenspan JR, Hill AP, Tyler CW (1979). Epidemiology of hepatocellular adenoma: the role of oral contraceptive use. JAMA.

[2] Reddy KR, Schiff ER (1993). Approach to a liver mass. Semin Liver Dis.

[3] Huurman VA, Schaapherder AF (2010). Management of ruptured hepatocellular adenoma. Dig Surg.

[4] Edmondson HA, Reynolds TB, Henderson B, Benton B (1977). Regression of liver cell adenomas associated with oral contraceptives. Ann Intern Med.

[5] van Aalten SM, Bröker ME, Busschbach JJ (2012). Pregnancy and liver adenoma management: PALM-study. BMC Gastroenterol.

[6] Wilson CH, Manas DM, French JJ (2011). Laparoscopic liver resection for hepatic adenoma in pregnancy. J Clin Gastroenterol.

[7] Sanford B, Hoeppner C, Ju T, Theisen BK, BuAbbud A, Estroff JM (2020). Multidisciplinary management of the pregnant patient in haemorrhagic shock secondary to an undiagnosed ruptured liver adenoma. BMJ Case Rep.

[8] Brolinson M, Tondo-Steele K, Chan M, Gable B (2019). Multidisciplinary in situ simulation to improve emergency obstetric care. BMJ Simul Technol Enhanc Learn.

[9] Rodehorst TK, Wilhelm SL, Jensen L (2005). Use of interdisciplinary simulation to understand perceptions of team members' roles. J Prof Nurs.

[10] Stroud J, Jenkins K, Bhandary S, Papadimos TJ (2017). Putting the pieces together: the role of multidisciplinary simulation in medical education. Symposium: simulation in medical education. Int J Acad Med.

[11] Patterson MD, Geis GL, LeMaster T, Wears RL (2013). Impact of multidisciplinary simulation-based training on patient safety in a paediatric emergency department. BMJ Qual Saf.

[12] Buzachero VV, Phillips J, Phillips P, Phillips ZL (2013). Measuring ROI in Healthcare: Tools and Techniques to Measure the Impact and ROI in Healthcare Improvement Projects and Programs.

[13] (2022). P value from T score calculator. https://www.socscistatistics.com/pvalues/tdistribution.aspx.

[14] Bullard MJ, Fox SM, Wares CM, Heffner AC, Stephens C, Rossi L (2019). Simulation-based interdisciplinary education improves intern attitudes and outlook toward colleagues in other disciplines. BMC Med Educ.

[15] Paige JT, Kozmenko V, Yang T, Paragi Gururaja R, Hilton CW, Cohn I Jr, Chauvin SW (2009). High-fidelity, simulation-based, interdisciplinary operating room team training at the point of care. Surgery.

[16] McGaghie WC, Barsuk JH, Wayne DB (2020). Comprehensive healthcare simulation: mastery learning in health professions education. Springer Nature.

[17] (2022). Common Program Requirements (residency) Contents Introduction. https://www.acgme.org/globalassets/PFAssets/ProgramRequirements/CPRResidency2021.pdf.

[18] Anderson LW, Krathwohl DR (2001). A taxonomy for learning, teaching, and assessing: a revision of Bloom’s taxonomy of educational objectives.

[19] Lichtenberger JP, Tatum PS, Gada S, Wyn M, Ho VB, Liacouras P (2018). Using 3D printing (additive manufacturing) to produce low-cost simulation models for medical training. Mil Med.

[20] Aimar A, Palermo A, Innocenti B (2019). The role of 3D printing in medical applications: a state of the art. J Healthc Eng.

